# Hyperuricemia Is Associated with Significant Liver Fibrosis in Subjects with Nonalcoholic Fatty Liver Disease, but Not in Subjects without It

**DOI:** 10.3390/jcm11051445

**Published:** 2022-03-07

**Authors:** Pei-Chia Yen, Yu-Tsung Chou, Chung-Hao Li, Zih-Jie Sun, Chih-Hsing Wu, Yin-Fan Chang, Feng-Hwa Lu, Yi-Ching Yang, Chih-Jen Chang, Jin-Shang Wu

**Affiliations:** 1 Department of Family Medicine, National Cheng Kung University Hospital, College of Medicine, National Cheng Kung University, Tainan 70403, Taiwan; sophieyen168@gmail.com (P.-C.Y.); odyssey4324@gmail.com (Y.-T.C.); sunzihjie@gmail.com (Z.-J.S.); paulo@mail.ncku.edu.tw (C.-H.W.); yinfan@mail.ncku.edu.tw (Y.-F.C.); fhlu@mail.ncku.edu.tw (F.-H.L.); yiching@mail.ncku.edu.tw (Y.-C.Y.); changcj.ncku@gmail.com (C.-J.C.); 2 Department of Health Management Center, National Cheng Kung University Hospital, College of Medicine, National Cheng Kung University, Tainan 70403, Taiwan; 3 Department of Family Medicine, An Nan Hospital, China Medical University, Tainan 70965, Taiwan; smallhear@gmail.com; 4 Health Examination Center, An Nan Hospital, China Medical University, Tainan 70965, Taiwan; 5 Department of Family Medicine, National Cheng Kung University Hospital Dou-Liou Branch, College of Medicine, National Cheng Kung University, Yunlin County 64043, Taiwan; 6 Department of Family Medicine, College of Medicine, National Cheng Kung University, Tainan 70101, Taiwan; 7 Institute of Geriatrics, College of Medicine, National Cheng Kung University, Tainan 70101, Taiwan; 8 Department of Family Medicine, Ditmanson Medical Foundation Chia-Yi Christian Hospital, Chiayi County 60002, Taiwan

**Keywords:** hyperuricemia, liver fibrosis, non-alcoholic fatty liver disease

## Abstract

Liver fibrosis is associated with liver-related outcomes, yet often remains underdiagnosed in primary care settings. Hyperuricemia is associated with non-alcoholic fatty liver disease (NAFLD), but the relationship between hyperuricemia and liver fibrosis remains unclear. Data on individuals without NAFLD is also limited. We investigated the association between hyperuricemia and liver fibrosis in subjects with and without NAFLD. This study recruited 11,690 relevant participants from a health-checkup center. NAFLD was based on ultrasonography. Hyperuricemia was defined as serum uric acid > 6.0 mg/dL in women and >7.0 mg/dL in men. Significant liver fibrosis was diagnosed with the aspartate aminotransferase to platelet ratio index ≥0.5. The following were positively associated with significant liver fibrosis: hyperuricemia (*p* = 0.001), age ≥ 65 years (*p* < 0.001), male gender (*p* < 0.001), obesity (*p* = 0.009), hypertension (*p* = 0.002), diabetes (*p* < 0.001), and NAFLD (*p* < 0.001) in the logistic regression. The positive association of hyperuricemia with significant liver fibrosis remained in subjects with NAFLD (*p* = 0.001), but not in subjects without NAFLD. In conclusion, hyperuricemia increased the associated risk of significant liver fibrosis. The positively associated risk existed in subjects with NAFLD, but not in those without it.

## 1. Introduction

Non-alcoholic fatty liver disease (NAFLD) affects more than 25% of the global population, being the most common cause of chronic liver disease both in Western countries and in Asia [1,2]. It has become a rising culprit leading to cirrhosis and even hepatocellular carcinoma [2]. Fibrosis stages are associated with liver-related outcomes and all-cause mortality in a dose-dependent manner [3]. However, liver fibrosis is often underdiagnosed in routine clinical practice, although early detection and management is important. The standard diagnosis is based on a biopsy, an invasive procedure with poor accessibility especially in primary care settings [2,4]. Noninvasive assessments for liver fibrosis have been developed, including biochemical serum markers, such as the aspartate aminotransferase (AST) to platelet ratio index (APRI), NAFLD fibrosis score (NFS), the Fibrosis-4 (FIB-4) Index, and the BARD score (body mass index (BMI), the AST/alanine aminotransferase (ALT) ratio, and diabetes mellitus), as well as image tests using transient elastography (FibroScan) or magnetic resonance elastography (MRE) [5]. However, MRE and FibroScan are expensive and have limited feasibility. Among various serum tests, the APRI has the advantage of using only two readily accessible primary care parameters while maintaining good ability to differentiate subjects with significant fibrosis from those without it [6,7,8,9,10].

Hyperuricemia is prevalent approximately 20% worldwide [11], having been reported to have associations with gouty arthritis, chronic kidney disease [12], metabolic syndrome, coronary heart disease [13], and early mortality [14]. In addition, hyperuricemia is known to be a marker for predicting NAFLD development [15,16,17], and acts as an independent risk factor for NAFLD progression to non-alcoholic steatohepatitis [18]. Studies with biopsy-proven NAFLD patients showed an association with hyperuricemia and the severity of histological liver damage and lobular inflammation [19]. However, the relationship between hyperuricemia and liver fibrosis is still inconsistent [20,21,22]. Moreover, most studies regarding hyperuricemia and liver fibrosis did not focus on subjects without NAFLD. Therefore, we aimed toward an exploration of the relationship between hyperuricemia and liver fibrosis in subjects with and without NAFLD.

## 2. Materials and Methods

### 2.1. Study Population

This retrospective cross-sectional study was conducted in the Health Examination Center at National Cheng Kung University Hospital (NCKUH), Tainan, Taiwan between June 2001 and December 2010. A total of 11,690 individuals participating in self-paid health examinations were included. Inclusion criteria were subjects aged ≥18 years with an abdominal sonography examination. Exclusion criteria were the following: (1) using anti-hyperuricemic agents, (2) liver cirrhosis, (3) suspected or confirmed liver malignancy, (4) hepatectomy by history or sonography, (5) viral hepatitis B and C based on serology or history, (6) history of alcoholic hepatitis or excessive alcohol use (>140 g/week) [23], (7) autoimmune, hemochromatosis, hereditary, Wilson’s disease, α1-antitrypsin deficiency or drug-related chronic liver disease, and (8) incomplete or missing data. Secondary data without personal identification were used and analyzed anonymously, so informed consent was waived. This study was approved by the Institutional Review Board, NCKUH (IRB Number: B-ER-108-326) in Tainan, Taiwan.

### 2.2. Clinical Measurements

All individuals filled out a structured questionnaire containing past medical histories, medication use, and lifestyle habits, which included cigarettes smoking, alcohol consumption, and regular exercise. Current smoking was defined as smoking at least 20 cigarettes per month for more than six months, and current drinking was defined as at least one drink per week for more than six months [24]. Habitual exercise was determined by vigorous exercise for a minimum of 20 min at least three times weekly [25,26].

BMI was defined as weight (kg)/square of height (m^2^), where those with BMI ≥ 27 kg/m^2^ were classified as obesity according to the Department of Health in Taiwan [27]. Blood pressure was measured after 5 min of rest with a DINAMAP vital sign monitor (Model 1846SX DINAMAP Monitor; Critikon Inc., Tampa, FL, USA). Hypertension was considered if there was a documented history of hypertension, use of an antihypertensive medication, right brachial systolic blood pressure (SBP) ≥ 140 mmHg or diastolic blood pressure (DBP) ≥ 90 mmHg [28]. All venous blood samples were collected after fasting for 10 h, and the laboratory tests included uric acid, cholesterol, triglyceride, high-density lipoprotein-cholesterol (HDL-C), fasting plasma glucose (FPG), glycated hemoglobin (Hba1c), creatinine, ALT, AST, and platelet count. A 2 h plasma glucose (2 h PG) specimen was collected 2 h after a 75 g oral glucose tolerance test. Hyperuricemia was defined as serum uric acid > 6.0 mg/dL in women and >7.0 mg/dL in men [29]. Hypercholesterolemia was defined as serum cholesterol levels ≥ 200 mg/dL, and hypertriglyceridemia was defined as triglyceride levels ≥ 150 mg/dL. Diabetes was determined by using diabetes history, taking antidiabetic medication, or meeting any of the three following diagnostic criteria proposed by the American Diabetes Association: (1) FPG ≥ 126 mg/dL, (2) Hba1c ≥ 6.5%, and (3) 2 h PG ≥ 200 mg/dL [30]. Abdominal sonographies (Xario SSA-660A; Toshiba, Nasu, Japan) were performed and interpreted by experienced radiologists. NAFLD was based on fatty liver shown by an ultrasonographic finding with decreases in lucidity, poor visualization of borders of the diaphragm and intrahepatic vessel walls, as well as increases in hepatic echogenicity or attenuation of the penetrated ultrasound signal [31]. The noninvasive APRI, which has been widely validated to determine significant liver fibrosis, was calculated as [(AST level/upper limit of normal of 40 U/L [32])/platelet count (10^3^/μL)] × 100. Participants were classified into two groups, where APRI greater than or equal to 0.5 was used to indicate significant liver fibrosis [8,9,33].

### 2.3. Statistically Analysis

SPSS software (version 17.0, SPSS, Inc., Chicago, IL, USA) was used for the data analysis. Continuous variables were presented as data means ± standard deviations, and categorical variables were presented as numbers (percentages). The significance of between-group differences in the continuous variables were tested using an independent student *t*-test, and differences in the categorical variables were evaluated using a Pearson’s chi-square analysis. Binominal logistic regressions were used to explore the association between hyperuricemia and significant liver fibrosis, as classified using the ARRI, with adjustment for age, gender, BMI, hypertension, hypercholesterolemia, hypertriglyceridemia, diabetes, current smoking, drinking, and habitual exercise in all subjects. Then, a subgroup analysis was performed on the subjects with and without NAFLD. Throughout the analyses, 2-sided *p* values of less than 0.05 were considered to be statistically significant.

## 3. Results

With 0.5 as the cutoff of APRI, 509 (4.4%) had significant liver fibrosis. All demographic and anthropometric details are presented in Table 1. Subjects with significant fibrosis were older and male-predominant, meanwhile having higher BMI, SBP, DBP, triglyceride, FPG, 2 h PG, Hba1c, creatinine, ALT, AST, uric acid, total bilirubin, alkaline phosphatase values than those without significant fibrosis. In addition, subjects with significant liver fibrosis had a higher prevalence of obesity, hypertension, hypercholesterolemia, hypertriglyceridemia, diabetes mellitus, hyperuricemia, and NAFLD, but a lower platelet counts and HDL-C than those without it.

Table 2 shows the odds ratio (OR) of the clinical variables for significant liver fibrosis after adjusting for other clinical variables. The following variables were positively associated with significant liver fibrosis: hyperuricemia (OR = 1.39, 95% CI: 1.15–1.69, *p* = 0.001), age ≥ 65 years (OR 2.45, 95% CI: 1.93–3.11, *p* < 0.001), male gender (OR 1.58, 95% CI: 1.27–1.96, *p* < 0.001), obesity (OR 1.32, 95% CI: 1.07–1.63, *p* = 0.009), hypertension (OR 1.40, 95% CI: 1.13–1.72, *p* = 0.002), diabetes (OR 1.88, 95% CI: 1.49–2.38, *p* < 0.001), and NAFLD (OR 2.37, 95% CI: 1.90–2.95, *p* < 0.001). To further determine the impact of NAFLD on the association between hyperuricemia and liver fibrosis, we did a subgroup analysis in subjects with and without NAFLD (Table 3). Hyperuricemia retained a positive association with significant liver fibrosis (OR 1.47, 95% CI: 1.17–1.85, *p* = 0.001) in the subjects with NAFLD, but not in the subjects without it. In both subjects with and without NAFLD, age ≥ 65 years, male gender, and having diabetes mellitus were positively associated with significant liver fibrosis. In addition, obesity, hypertension, and habitual exercise were found to be independently associated with significant liver fibrosis in the subjects with NAFLD. In the subjects without NAFLD, hypertriglyceridemia led to an increased odds of significant liver fibrosis.

## 4. Discussion

This cross-sectional study revealed 509 individuals (4.4%) with significant liver fibrosis in a population aged ≥ 18 years, which is compatible with the prevalence of 3.6~5.8% found in a general population in Europe [34]. Our results showed hyperuricemia was positively associated with significant liver fibrosis after adjusting for age, sex, BMI, hypertension, hypercholesterolemia, hypertriglyceridemia, diabetes, NAFLD, current smoking, drinking, and habitual exercise. In addition, the positive association was maintained in the subgroup with NAFLD, but not in those without it. Two studies [35,36] showed a positive relationship between hyperuricemia and liver fibrosis; five presented neutral results [17,19,37,38,39], and one found an inverse association [40]. These discrepancies may be attributed to different definitions of liver fibrosis and hyperuricemia, the subjects’ inclusion and exclusion criteria, and adjustment for confounding factors. Using noninvasive tests instead of the gold standard-liver biopsy for liver fibrosis is important for early detection in routine clinical practice. The APRI, contained with AST ratios and platelet counts, has similar sensitivity and specificity to that of the BARD, the FIB-4 Index, and the NFS [41], while being simple and convenient in primary care settings. Younossi found that the APRI performed better in terms of identifying patients with and without liver stiffness ≥ 8 kPa, because their APRI were significantly different (0.665 vs. 0.316, *p* < 0.05), while FIB-4 Index and NFS scores were not [42]. Furthermore, as compared to the APRI, the FIB-4 and NFS had lower accuracy regarding liver fibrosis diagnoses in subjects ≥ 65 years old [43]. In addition, some previous studies had identical definition of hyperuricemia for both men and women [17,35]. As compared to the general population, some studies included only participants with elevated liver enzymes [17,19,40]. One study in Indonesia excluded subjects with any history of alcohol consumption [36], so their results limited generalization in the real world. Many studies included individuals taking hyperuricemia agents [17,19,21,35,36,37,38,39,40], which may have led to misclassification of hyperuricemia. Five studies included patients with cirrhosis [17,35,36,37,38], which may interfere the production of uric acid [35,44]. Studies conducted by Sertoglu [17] and Ballestri [39] excluded patients with hypertension or diabetes, which is common in clinical practice and presents as an independent risk factor for liver fibrosis [35]. In addition, important lifestyle factors, including current smoking, current alcohol consumption, and habitual exercise were not considered in previous studies [35,36,38,39,40], and some studies did not adjust for sex [38], age [36], BMI [35,36,37,38], serum glucose status, or dyslipidemia [38]. To the best of our knowledge, this is the first study to show that hyperuricemia is associated with significant liver fibrosis, especially in subjects with NAFLD, after excluding subjects taking anti-hyperuricemic agents and those with existing liver disorders and carefully adjusting for important confounding factors, including age, sex, BMI, hypertension, hypercholesterolemia, hypertriglyceridemia, diabetes, current smoking, current alcohol consumption, and habitual exercise.

The mechanism of the positive relationship between hyperuricemia and significant liver fibrosis is still not well understood. Insulin resistance [35], macrophage and inflammasome activation, increased ROS (reactive oxygen species), and decreased nitric oxide may offer some possible explanations [18,19], but further studies are needed. First, hyperuricemia increases the expression of monocyte chemoattractant protein-1, which leads to migration and infiltration of macrophages and activates the nucleotide-binding oligomerization domain-LRR- and pyrin domain-containing protein 3 inflammasomes through secretion of pro-inflammatory cytokines, such as interleukin-1β and interleukin-18. Both of these mechanisms induce hepatocyte necrosis and apoptosis [36,44,45]. Second, the triggered ROS environment stimulates profibrogenesis mediators from Kupffer cells and inflammatory cells, which eventually causes activation of hepatic stellate cells [46], leading to accumulation of excess extracellular matrices and initiation of the fibrosis process [20,47]. Xanthine oxidase, the main enzyme necessary for metabolizing uric acid, may also enhance superoxide production under circumstances with overloading of purine bodies [18]. Third, uric acid decreases nitric oxide production and drives liver sinusoid endothelial dysfunctions, which also activates hepatic stellate cells and impairs liver cell regeneration [19,48]. In this study, we found a positive association between hyperuricemia and significant liver fibrosis in subjects with NAFLD, but not in those without it. The explanation for this difference is not clear. One possible explanation might be related to the pro-oxidant ability of uric acid in hydrophobic but not hydrophilic environments through activation of nicotinamide adenine dinucleotide phosphate oxidase and increasing intracellular ROS [49]. Under circumstances involving excessive lipid disposition, uric acid is an external contributing toxic factor that aggravates lipotoxicity and increases the endoplasmic reticulum and oxidant stress, all of which accelerate hepatocyte necroptosis [44]. On the contrary, in subjects without NAFLD, uric acid may not serve as a pro-inflammatory mediator because free fatty acid enters well-functioned mitochondria for beta-oxidation, since there is no overwhelming disposal of free fatty acid [44].

In this study, older adults, male gender, obesity, hypertension, and diabetes were independently related to significant liver fibrosis. Increased age was associated with higher prevalence of a biopsy-proven higher fibrosis stage [43], which was due to modified vascular morphology and decreased arteriolar diameters, which cause sinusoidal fibrosis and impaired oxygen-dependent hepatocyte functioning [50,51]. Evidence of an insignificant association in previous studies could have been due to narrow age distribution in the study population [19,35]. Similar to this study, some studies showed male gender was either a predictor for advanced NAFLD [3] or an independent risk factor for liver fibrosis [34,52]; however, some did not [19,35]. One possible explanation for this conflicting finding is the protective role of estrogen, which blocks proliferation and fibrogenesis by stellate cells in females [53]. In line with the previous literature [3,54], a higher BMI and obesity were associated with progression of liver fibrosis and elevated liver stiffness throughout insulin resistance [35] and endocrine imbalance in the adipose tissue [19]. A study in Turkey [17] showed an insignificant association between BMI and liver fibrosis. Possible reasons for this finding were the fact that all the subjects were of male, and a majority of them were obese. There were also ethnic differences. A higher prevalence of advanced fibrosis was found in subjects with hypertension [55]. Hypertension may induce liver regeneration and fibrosis through an endothelial dysfunction due to increased angiocrine signals [56] and may also be triggered by glucose intolerance and decreased interleukin-10-mediated or heme oxygenase-1-induced anti-inflammatory mechanisms [57]. Evidence of an increased associated risk of liver fibrosis with diabetes [19,34,35,39,52,53] had been consistent. In 2019, the American Diabetes Association recommended evaluation of fibrosis in patients with type 2 diabetes with steatosis or elevated ALT [58]. Though not clearly established, the proposed mechanisms were insulin resistance with adipocyte dysfunction, which increases mitochondrial stress and adipokines, leading to activation of hepatic stellate cells [35,59].

Despite the large number of participants with careful subject selection and adjustments for confounding factors, this study does have some important limitations. First, it was a cross-sectional design. Determining a causal relationship between hyperuricemia and significant liver fibrosis thus requires further studies. Second, we used the serum biomarker APRI as the surrogate for liver fibrosis instead of pathological report from a biopsy, which is the gold standard, or more sensitive image tests such as an MRE or FibroScan. Unfortunately, FibroScan was not available in this study. However, APRI score could be useful in the diagnosis of fibrosis in the primary care, as it appears to increase with a higher METAVIR score (biopsy scale for liver fibrosis) and its AUROCs for predicting significant fibrosis were 0.87 (95% CI, 0.79–0.95) [60,61]. In addition, one European real-world article revealed that patients with liver fibrosis based on transient elastography (liver stiffness ≥ 8 kPa) have higher APRI (0.665 vs. 0.316) [42]. Furthermore, AST and platelet count are relatively obtainable that often included in routine checkups. For screening purposes in a large asymptomatic community-dwelling population, it is still a useful tool for primary risk stratification. In the meantime, improving the efficiency of referrals to specialists will lead to avoidance of unnecessary complications after biopsy. This study did not exclude participants with both normal ALT and AST based on the consideration of extrapolation to the primary care situation [62]. In the group of significant fibrosis, 19.1% (97/509) had both normal ALT and AST. The prevalence of significant fibrosis was 1% (97/9614) in individuals with normal ALT and AST. Third, instead of using quantitative measurement of steatosis, such as controlled attenuation parameter, we used ultrasound for NAFLD diagnosis, which were more reliable when more than 33% of hepatocytes are steotatic. However, ultrasound bares high diagnostic accuracy and had been strongly recommended as a first-line image in patients with suspected NAFLD [60]. Fourth, we were not able to obtain participants’ portion of high-purine diet, which were largely related to hyperuricemia. We also relied largely on documented liver diseases, such as autoimmune, hemochromatosis, hereditary, Wilson’s disease, α1-antitrypsin deficiency, or drug-related chronic liver disease, instead of acquiring accurate lab diagnosis. Fifth, our participants were limited to a population of Taiwanese individuals, so applicability to other ethnicities requires more studies. Finally, we were unable to provide genomic biomarkers and microbiome information, which have some role in liver fibrosis [5].

## 5. Conclusions

In conclusion, hyperuricemia was shown to be positively associated with significant liver fibrosis. The positive association was found in subjects with NAFLD, but not in those without NAFLD. In addition, older adults, male gender, obesity, hypertension, and diabetes also increased the associated risk of significant liver fibrosis.

## Figures and Tables

**Table 1:** Comparisons of clinical parameters in subjects with and without significant liver fibrosis.

	Significant Liver Fibrosis		
	No	Yes	
	N = 11,181	N = 509	*p* Value
Age, years	48.0 ± 12.6	53.2 ± 14.0	<0.001
Age ≥ 65 years	1028 (9.2%)	113 (22.2%)	<0.001
Male	6414 (57.4%)	371 (72.9%)	<0.001
BMI, kg/m^2^	24.1 ± 3.5	26.1 ± 4.1	<0.001
Obesity (BMI ≥ 27 kg/m^2^)	2066 (18.5%)	187 (36.7%)	<0.001
Systolic BP, mmHg	117.0 ± 17.0	127.3 ± 18.9	<0.001
Diastolic BP, mmHg	69.2 ± 10.8	74.9 ± 12.0	<0.001
Hypertension	1843 (16.5%)	164 (32.2%)	<0.001
Cholesterol, mg/dL	198.1 ± 37.1	201.4 ± 38.8	0.056
Cholesterol ≥ 200 mg/dL	5059 (45.2%)	277 (54.4%)	<0.001
Triglyceride, mg/dL	129.8 ± 90.9	175.4 ± 141.7	<0.001
Triglyceride ≥ 150 mg/dL	3048 (27.3 %)	225 (44.2%)	<0.001
HDL-C, mg/dL	49.6 ± 13.6	45.6 ± 14.9	<0.001
FPG, mg/dL	90.8 ± 17.3	100.4 ± 31.4	<0.001
2 h PG, mg/dL	122.2 ± 51.3	158.6 ± 79.2	<0.001
Hba1c, %	5.6 ± 0.7	6.0 ± 1.2	<0.001
Diabetes mellitus	930 (8.3%)	115 (22.6%)	<0.001
Creatinine, mg/dL	0.88 ± 0.38	0.94 ± 0.46	0.004
ALT, U/L	27.2 ± 16.5	81.0 ± 58.2	<0.001
AST, U/L	23.5 ± 7.0	58.6 ± 42.8	<0.001
Uric acid, mg/dL	6.0 ± 1.5	6.7 ± 1.7	<0.001
Hyperuricemia	3164 (28.3%)	235 (46.2%)	<0.001
Platelet, 10^3^/μL	255.8 ± 58.8	195.9 ± 59.4	<0.001
NAFLD	7318 (34.5%)	336 (66.0%)	<0.001
APRI	0.28 ± 0.17	1.07 ± 1.15	<0.001
Total bilirubin, mg/dL	0.8 ± 0.4	1.0 ± 0.6	<0.001
Albumin, g/dL	4.4 ± 0.3	4.4 ± 0.4	0.314
Alkaline Phosphatase, U/L	63.3 ± 19.6	71.3 ± 26.7	<0.001
Current smoking	1211 (10.8 %)	64 (12.6%)	0.215
Current drinking	1235 (11.0 %)	69 (13.6 %)	0.081
Habitual exercise	909 (8.1%)	31 (6.1%)	0.117

Data are presented as the mean ± standard deviation or numbers (percent); BMI: body mass index; BP: blood pressure; HDL-C: high-density lipoprotein-cholesterol; FPG: fasting plasma glucose; 2 h PG: 2 h plasma glucose; A1C: glycated hemoglobin; ALT: alanine aminotransferase; AST: aspartate transaminase; NAFLD: non-alcoholic fatty liver disease; APRI: the AST to Platelet Ratio Index, calculated as AST/40/Platelet (10^3^/μL) ∗ 100.

**Table 2:** Binomial logistic regression for the relationship between clinical variables and significant liver fibrosis.

	OR (95% CI)	*p* Value
Age ≥ 65 years, yes vs. no	2.45 (1.93–3.11)	<0.001
Male vs. female	1.58 (1.27–1.96)	<0.001
Obesity, yes vs. no	1.32 (1.07–1.63)	0.009
Hypertension, yes vs. no	1.40 (1.13–1.72)	0.002
Cholesterol ≥ 200 mg/dL yes vs. no	1.11 (0.92–1.34)	0.271
Triglyceride ≥ 150 mg/dL, yes vs. no	1.15 (0.94–1.41)	0.176
Diabetes, yes vs. no	1.88 (1.49–2.38)	<0.001
Hyperuricemia, yes vs. no	1.39 (1.15–1.69)	0.001
NAFLD, yes vs. no	2.37 (1.90–2.95)	<0.001
Current smoking, yes vs. no	1.00 (0.74–1.34)	0.989
Current drinking, yes vs. no	1.03 (0.77–1.37)	0.859
Habitual exercise, yes vs. no	0.78 (0.53–1.14)	0.193

OR: odds ratio; CI: confidence interval; BMI: body mass index. NAFLD; non-alcoholic fatty liver disease.

**Table 3:** Binomial logistic regression of the relationship between clinical variables and significant liver fibrosis in subjects with and without NAFLD.

	NAFLD (+) N = 4199		NAFLD (−) N = 7401	
	OR (95% CI)	*p* Value	OR (95% CI)	*p* Value
Age ≥ 65 years, yes vs. no	1.79 (1.29–2.47)	<0.001	3.98 (2.75–5.74)	<0.001
Male vs. female	1.61 (1.21–2.14)	0.001	1.44 (1.03–2.01)	0.032
Obesity, yes vs. no	1.37 (1.09–1.72)	0.008	1.00 (0.57–1.74)	0.997
Hypertension, yes vs. no	1.43 (1.12–1.83)	0.004	1.19 (0.80–1.76)	0.397
Cholesterol ≥ 200 mg/dL, yes vs. no	1.26 (0.99–1.59)	0.055	0.87 (0.63–1.19)	0.382
Triglyceride ≥ 150 mg/dL, yes vs. no	0.96 (0.76–1.21)	0.707	1.82 (1.26–2.62)	0.001
Diabetes, yes vs. no	1.84 (1.41–2.41)	<0.001	2.18 (1.38–3.46)	0.001
Hyperuricemia, yes vs. no	1.47 (1.17–1.85)	0.001	1.23 (0.86–1.74)	0.256
Current smoking, yes vs. no	1.21 (0.86–1.69)	0.280	0.59 (0.32–1.11)	0.102
Current drinking, yes vs. no	1.00 (0.71–1.41)	0.985	1.12 (0.67–1.88)	0.656
Habitual exercise, yes vs. no	0.53 (0.31–0.93)	0.027	1.22 (0.73–2.05)	0.444

OR: odds ratio; CI: confidence interval; BMI: body mass index. NAFLD; non-alcoholic fatty liver disease.

## Data Availability

The data presented in this study are available on reasonable request from the corresponding author.
